# Transversus Abdominis Plane Block Appears to Be Effective and Safe as a Part of Multimodal Analgesia in Bariatric Surgery: a Meta-analysis and Systematic Review of Randomized Controlled Trials

**DOI:** 10.1007/s11695-020-04973-8

**Published:** 2020-10-21

**Authors:** Mária Földi, Alexandra Soós, Péter Hegyi, Szabolcs Kiss, Zsolt Szakács, Margit Solymár, Erika Pétervári, Márta Balaskó, Krzysztof Kusza, Zsolt Molnár

**Affiliations:** 1 Institute for Translational Medicine, Medical School, University of Pécs, 12 Ifjúság St., Pécs, 7624 Hungary; 2 Szentágothai Research Centre, University of Pécs, Pécs, Hungary; 3 Doctoral School of Clinical Medicine, University of Szeged, Szeged, Hungary; 4 Division of Translational Medicine, First Department of Medicine, Medical School, University of Pécs, Pécs, Hungary; 5 Department of Anesthesiology and Intensive Therapy, Poznan University for Medical Sciences, Poznan, Poland

**Keywords:** Pain, Bariatric surgery, TAP block, Meta-analysis

## Abstract

**Purpose:**

Pain after bariatric surgery can prolong recovery. This patient group is highly susceptible to opioid-related side effects. Enhanced Recovery After Surgery guidelines strongly recommend the administration of multimodal medications to reduce narcotic consumption. However, the role of ultrasound-guided transversus abdominis plane (USG-TAP) block in multimodal analgesia of weight loss surgeries remains controversial.

**Materials and Methods:**

A systematic search was performed in four databases for studies published up to September 2019. We considered randomized controlled trials that assessed the efficacy of perioperative USG-TAP block as a part of multimodal analgesia in patients with laparoscopic bariatric surgery.

**Results:**

Eight studies (525 patients) were included in the meta-analysis. Pooled analysis showed lower pain scores with USG-TAP block at every evaluated time point and lower opioid requirement in the USG-TAP block group (weighted mean difference (WMD) = − 7.59 mg; 95% CI − 9.86, − 5.39; *p* < 0.001). Time to ambulate was shorter with USG-TAP block (WMD = − 2.22 h; 95% CI − 3.89, − 0.56; *p* = 0.009). This intervention also seemed to be safe: only three non-severe complications with USG-TAP block were reported in the included studies.

**Conclusion:**

Our results may support the incorporation of USG-TAP block into multimodal analgesia regimens of ERAS protocols for bariatric surgery.

**Electronic supplementary material:**

The online version of this article (10.1007/s11695-020-04973-8) contains supplementary material, which is available to authorized users.

## Introduction

Pain in the postoperative period can cause serious suffering to patients, prolong recovery, and increase healthcare costs [1]. However, postoperative pain management can be a major challenge as previous studies demonstrated that it is frequently suboptimal [2–4].

Laparoscopic bariatric surgeries are considered minimally invasive, but they can cause severe pain [5, 6]. Opioids are excellent analgesics, but they have several side effects such as respiratory depression, which may further complicate pain management in weight loss surgeries, particularly in cases with obstructive sleep apnea [7]. Other comorbidities such as diabetes mellitus and cardiovascular diseases that are common in patients with obesity can also lead to difficulties with pain management [8]. This complexity highlights the importance and the challenges of the optimal choice of analgesia in bariatric surgery.

Enhanced Recovery After Surgery (ERAS) protocols are created to facilitate faster recovery after surgery multimodal analgesia [9]. Although growing evidence supports multimodal analgesic techniques in clinical practice, opioids still remain among the first choice of postoperative pain management [10].

Postoperative opioid overuse could be particularly worrisome. For example, in the USA, the opioid epidemic causes a serious health crisis. According to a recent study, persistent opioid use is a common problem after surgery [11]. In the opioid epidemic era, recognizing the issue of opioid overuse with its associated complications could be of particular importance [12]. Several alternative options can be used including other pain medications such as paracetamol, non-steroidal anti-inflammatory drugs (NSAIDs), ketamine, or gabapentin [13].

Besides pharmacological analgesia, locoregional analgesic techniques are also among the alternatives. After decades of being the “gold standard,” large meta-analyses and trials reported controversial effects of epidural analgesia on mortality and morbidity associated with frequent technical failures [14, 15]. As an alternative to epidural analgesia, infiltrative techniques—including transversus abdominis plane block (TAP block)—has gained increasing attention in recent years as they can be safely and easily applied [16]. During TAP block, a local anesthetic solution is injected between planes of abdominal muscles to anesthetize the anterior abdominal wall [17]. As ultrasound guidance (USG) becomes more widely available, the popularity of TAP block has further increased. USG facilitates the performance of TAP block in cases where anatomic landmarks are poorly defined, e.g., in patients with obesity [18].

Recent meta-analyses showed that USG-TAP block is effective in reducing pain and opioid consumption in different abdominal surgeries [19], including open appendectomy [20], hysterectomy [21], or colorectal resection [15] to control pain and decrease opioid consumption. Randomized controlled studies (RCTs) investigating the use of TAP block in weight loss surgeries have also been published, but its impact on different outcomes remained controversial. To our knowledge, no meta-analysis has examined TAP block during laparoscopic bariatric surgery. Therefore, we aimed to assess the effects of USG-TAP block as a part of multimodal analgesia for postoperative pain management in patients undergoing laparoscopic bariatric surgery.

## Methods and Materials

We report this systematic review and meta-analysis following the Preferred Reporting in Systematic Reviews and Meta-analyses (PRISMA) (Supplementary Material) [22]. We registered the protocol on PROSPERO under registration number CRD42020154384.

### Eligibility Criteria

We included full-text RCTs that assessed the efficacy of perioperative USG-TAP block in postoperative analgesia compared with no treatment or sham intervention in patients who underwent laparoscopic bariatric surgery.

The following outcomes were analyzed: pain scores measured by the Visual Analog Scale (VAS) or the Numbering Rating Scale (NRS) on a scale from 0 to 10 within the first 24 postoperative hours, morphine requirement (mg) within the first 24 postoperative hours, rate of nausea during phase I recovery, time to ambulate (hours), length of hospital stay (hours), operation time (hours).

### Search Strategy

A systematic search was carried out in the following databases for studies published up to September 2019: CENTRAL, MEDLINE, Web of Science, and Embase. We designed a search key with synonyms to bariatric surgery (population) and TAP (intervention) linked with Boolean operators. We did not use any filters (e.g., language, full-text, human) (Supplementary Material). The reference lists of included studies and previous systematic reviews and meta-analyses have also been screened for additional articles. Gray literature was not included in our meta-analyses.

### Selection Strategy and Data Extraction

Two authors independently (SK and MF) removed all duplicate records, then checked titles and abstracts to remove irrelevant articles, and evaluated full-text articles, whether they were eligible for inclusion. All disagreements were resolved by consensus.

Two authors independently (MF and SK) extracted data into a standardized data collection sheet. We resolved any disagreement by consensus. From the individual studies, we extracted the raw data (mean and standard deviation or standard error) in case of cumulative morphine dose, time to ambulate, length of hospital stay, operation time, and pain level in rest and at movement if it was given. In the case of nausea, the number of patients and event rates in the two groups were extracted from the individual studies.

### Risk of Bias Assessment

Two independent authors (MF and SK) used the revised Cochrane risk-of-bias 2 (RoB 2) tool to assess the risk of bias of studies in the following categories [23]. Disagreements were resolved by consensus.

### Statistical Analysis

We calculated mean differences with 95% CI between the control and USG-TAP groups. In the case of nausea, we calculated risk ratio with 95% CI. A *p* value < 0.05 was considered statistically significant. Pooled estimates were calculated with a random effects model by using the DerSimonian-Laird method [24]. If mean with standard deviation was not reported, we estimated them from median, interquartile, and range [25]. Results of the meta-analysis were displayed graphically using forest plots. Due to methodological characteristics of the analysis, we could not indicate pooled means for each group on the forest plots; however, study-level data in each study can be seen in Supplementary Material (for 24-h cumulative morphine requirement, time to ambulate, length of hospital stay, and operation time).

Heterogeneity was tested by using the Cochrane’s *Q* and the *I*^2^ statistics, where *I*^2^ = 100% × (*Q* − df) / *Q*, and represents the magnitude of the heterogeneity (moderate: 30–60%, substantial: 50–90%, considerable: 75–100%) [16]. A *p* value < 0.10 was considered statistically significant heterogeneity. All meta-analytical calculations were performed by Stata 11 data analysis and statistical software (Stata Corp LLC, College Station, TX, USA).

We performed trial sequential analysis (TSA) for each outcome if it was possible. We used the TSA tool to estimate the required number of patients in future studies and to quantify the statistical reliability of data if the condition of the tests were met. With this test, we assessed whether the intervention arm is effective applying adjusted significance tests and determined the necessity of conducting more studies in the topic to show significant differences [26].

We planned to conduct the following subgroup analyses: gender, age, type of bariatric surgery, type and dose of local anesthetics, TAP approach. Because of the limited number of studies, we were unable to conduct any of the planned subgroup analyses.

### Quality of Evidence

We assessed the overall quality of evidence using the GRADE profiler (GRADEpro). Since data come from only RCTs, we downgraded the evidence from “high quality” by one level for serious (or by two levels for very serious) risk of bias, indirectness of evidence, serious inconsistency, imprecision of effect estimates, or potential publication bias.

We included the critical and important outcomes in the “Summary of findings table” (Table 2).

## Results

### Results of Search and Selection

**Fig. 1:**
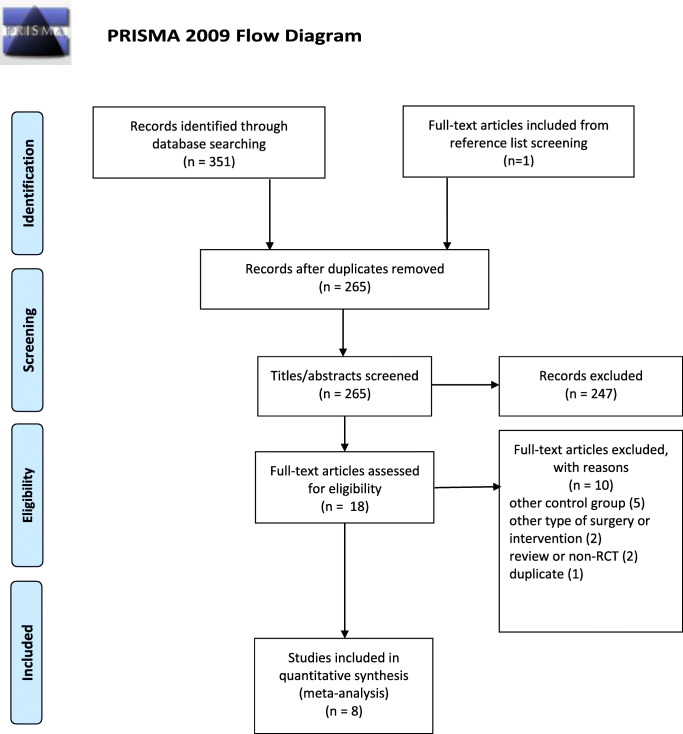
Flow chart of study selection and inclusion process

The selection process is described in detail in the PRISMA flow diagram (Fig. 1). A total of 351 records were identified through electronic database search (CENTRAL: 89; MEDLINE: 36; Web of Science: 99; Embase 127), eight of which were included in this meta-analysis (*n* = 525; 262 in the “USG-TAP block” group and 263 in the “control” group). Beyond the eight analyzed articles, two studies with active control groups were excluded [27, 28], and in one excluded study, USG-TAP was not performed perioperatively [29].

### Characteristics of the Studies Included

All included studies were single-center RCTs (Table 1). From the eight studies, five used sham-control (normal saline infiltration) [30–34]. In three studies, the control group did not receive sham-control [35–37]. One study used port-site infiltration in both intervention and control groups [37].

**Table 1:** The “Characteristics of included studies” table

Study name	Country/setting	Allocation	Participants	Characteristics of participants	Type of surgery	Type and dose of local anesthetic agent	TAP approach	Outcomes	Postoperative analgesia regimen (PACU)
Albrecht 2013	Single center in Canada	USG-TAP	27	Mean age 44.8 (95% CI, 40.8–48.8), 74% female, mean BMI 49.3 (95% CI 45.6–52.9)	Lap. gastric bypass surgery	20 mL of 0.25% bupivacaine	Preop. oblique subcostal	24-h cumulative opioid consumption, length of hospitalization, rate of nausea and vomiting	As needed with incremental doses of fentanyl 25–50 μg iv and morphine 1–2 mg iv or hydromorphone 0.2–0.4 mg iv in order to achieve a clinical target of 4/10 or lower on a Numbering Rating Scale (NRS) for pain
		No USG-TAP*	30	Mean age 38.8 (95% CI, 34.9–42.8), 87% female, mean BMI 48.9 (95% CI, 49.5–51.8)					
De Oliveira 2014	Single center in the USA	USG-TAP	9	Median age 47.0 (39–53), 80% female, median BMI 44.2 (39.0–45.7)	Lap. gastric band surgery	20 mL of 0.5% ropivacaine	Preop. posterior	24-h cumulative opioid consumption, length of hospitalization, rate of nausea and vomiting, operation time	As needed with hydromorphone 0.4 mg iv to achieve 4/10 or lower on a Numbering Rating Scale (NRS) for pain. When oral medications were tolerated, hydrocodone 10 mg plus acetaminophen 325 mg
		Sham	10	Median age 50.0 (36–54), 78% female, median BMI 40.1 (39.0–45.7)					
Emile 2019	Single center in Egypt	USG-TAP	46	Mean age 35.8 ± 8.9, 94% female, mean BMI 50.4 ± 7.9	Lap. bariatric surgery	20 mL of 0.25% bupivacaine	Postop. mid-axillary	Pain scores at 1, 6, 12, and 24 h at rest, time to ambulate, length of hospitalization	Paracetamol (1 g every 8 h) iv. As needed with 0.2 mg/kg pethidine iv in order to achieve a clinical target of 4/10 or lower on a Visual Analog Scale (VAS) for pain
		No USG-TAP*	46	Mean age 33.6 ± 9.8, 91% female, mean 48.6 ± 5.3					
Ibrahim 2014	Single center in Egypt	USG-TAP	21	Mean age 38.3 ± 10.2, 76% female, mean BMI 48.5 ± 10.4	Lap. gastrectomy	30 mL of 0.25% bupivacaine	Preop. oblique subcostal	24-h cumulative opioid dose, operation time	As needed with fentanyl 25–50 μg iv or morphine 1–2 mg iv or pethidine 20–40 mg iv if patient had moderate or severe pain
		Sham	21	Mean age 37.4 ± 11.3, 68% female, mean BMI 46.4 ± 8.7					
Mittal 2018	Single center in India	USG-TAP	30	Mean BMI 46.2 ± 6.7	Lap. sleeve gastrectomy	40 mL of 0.375% ropivacaine	Preop. mid-axillary	Pain scores at 1, 3, 6, 12, and 24 h at rest, time to ambulate	Diclofenac (75 mg every 8 h) iv. As needed with 1 g diclofenac iv in order to achieve a clinical target of 4/10 or lower on a Visual Analog Scale (VAS) for pain
		No USG-TAP*	30	Mean BMI 44.9 ± 7.2					
Saber 2018	Single center in Canada	USG-TAP	30	Mean age 37.0 ± 10.7, 87% female, mean BMI 44.0 ± 4.8	LAP. sleeve gastrectomy	20 mL of 0.25% bupivacaine	Preop. oblique subcostal	Pain scores at 3 h at rest, operation time	Acetaminophen 600 mg q6, gabapentin 100 mg. As needed with morphine and hydromorphone
		Sham	30	Mean age 40.0 ± 11.2, 94% female, mean BMI 44.0 ± 7.1					
Sherif 2015	Single center in Egypt	USG-TAP	48	Mean age 40.9 ± 8.75, 21% female, mean BMI 38.7 + 2.2	Lap. gastric bypass	20 mL of 0.5% bupivacaine	Postop. anterior axillary	Pain scores at 1, 6, 12, and 24 h at rest, 24-h cumulative opioid dose, time to ambulate, rate of nausea and vomiting	Intravenous patient-controlled analgesia (PCA) system, which provided 1 mg of morphine on demand with a block-out interval of 20 min and a maximum 6 h dose of 10 mg in both groups. All patients received regular postoperative analgesia comprising paracetamol 1 g, intravenous, four times daily
		Sham	47	Mean age 40.4 ± 8.71, 26% female, mean BMI 38.9 ± 2.2					
Sinha 2013	Single center in India	USG-TAP	50	Mean age 39.9 ± 13.3, mean BMI 48.1 ± 6.3	Lap. gastric bypass	20 mL of 0.375% bupivacaine	Postop. oblique subcostal	Pain scores at 1, 3, 6, 12, and 24 h at rest, time to ambulate	Tramadol
		Sham	50	Mean age 39.1 ± 10.6, mean BMI 45.6 ± 6.6					

no USG-TAP* indicates no sham-control was applied

*USG-TAP* ultrasound-guided transversus abdominis plane, *lap.* laparoscopic, *preop.* pre-operative, *postop.* postoperative

Comments: Patients always received standard medical therapy, including pain management (non-opioids and opioids), antiemetics, antibiotics, thromboprophylaxis, if necessary

Data are expressed either as mean ± SE/SD, as median (interquartile range), or as mean (95% confidence interval)

Studies reported data of patient group numbers ranging from 19 to 100. Studies enrolled predominantly women with a mean BMI over 40 [33]. Four studies reported data of patients undergoing laparoscopic sleeve gastrectomy [31–33, 36]. Two studies recruited patients who underwent laparoscopic gastric bypass surgery [34, 37]. One trial studied patients with gastric band surgery [30] and one with several different types of laparoscopic bariatric surgery [35].

The type and dose of local anesthetic agents and those of USG-TAP approaches were different among studies. In four of the studies, USG-TAP block was performed immediately after completion of surgery [30–32, 37]; the remaining studies carried out surgeries with preoperative USG-TAP block after anesthesia induction [33–36].

Postoperative analgesia regimens were also quite diverse among studies (see in detail in Table 1); most of the studies used regular or as-needed non-opioids supplemented with narcotics on demand. However, some studies—carried out in the early 2010—applied opioids exclusively [31, 34].

### Effects of Intervention

#### Primary Endpoints

##### Pain Scores Within the First 48 h

**Fig. 2:**
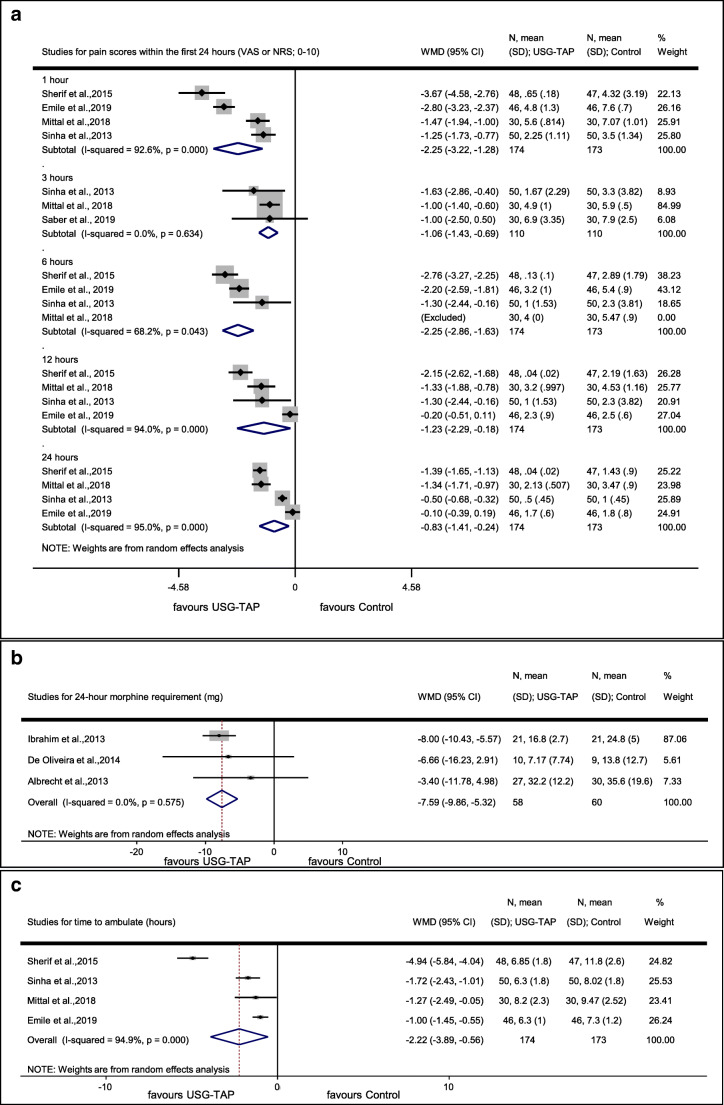
Forest plots that show efficacy endpoints for the comparison of “USG-TAP” and “control”. **a** Forest plot for pain score within the first 24 postoperative hours (VAS or NRS, 0–10). **b** Forest plot showing 24-h postoperative morphine requirement (mg). **c** Forest plot showing time to ambulate (h). USG-TAP, ultrasound-guided transversus abdominis plane block; VAS, Visual Analog Scale; NRS, Numbering Rating Scale

Pooled analysis showed that USG-TAP block lowered postoperative pain scores (rated on a scale between 0 and 10) at rest by 2.25 (*p* < 0.001) at 1 h, by 1.08 (*p* < 0.001) at 3 h, by 2.25 (*p* < 0.001) at 6 h, by 1.23 (*p* < 0.022) at 12 h, and by 0.83 (*p* = 0.006) at 24 h (Fig. 2a). Heterogeneity was considerable in these analyses (Fig. 2a).

Two studies also examined pain scores at rest 48 h after surgery: they found significantly lower pain scores in the USG-TAP block group [33, 36].

In two included studies [33, 36], pain scores at movement were also significantly lower at each evaluated time point (0.5, 3, 6, 12, 24, and 48 h postoperatively; *p* < 0.001 for all comparisons).

##### Postoperative Cumulative Morphine Dose

Four studies with 213 patients (106 in the intervention group and 107 in the control group) examined the postoperative cumulative morphine dose within the first 24 h [30, 31, 33, 37]. Morphine requirement did not differ significantly between the intervention and control groups (− 12 mg; 95% CI − 26.88, 2.89; *p* = 0.114). However, we observed high heterogeneity in this analysis (*p*_heterogeneity_ *<* 0.001 and *I*^2^ = 99.0%). We identified and removed the influential study with sensitivity analysis, which reduced heterogeneity to 0% and changed a direction of the main association to favoring TAP (Fig. 2b) [33]. Results of each study can be seen in Supplementary Material.

#### Secondary Endpoints

##### Time to Postoperative Bowel Recovery

One trial with 46 patients in each arm reported recovery of bowel functions assessed by time to first flatus, and they found a statistically significant difference favoring the USG-TAP block group (9.5 ± 1.9 vs 10.5 ± 2.2 h; *p* < 0.001) [35]. Mittal and coworkers also found earlier resumption of bowel activity in the intervention group [36].

##### Nausea and Vomiting

Pooled analyses of three studies with 171 patients (85 in the intervention and 86 in the control groups) indicated a lower risk of nausea in the USG-TAP block groups compared with control patients (95% CI, RR = 0.24, *p* < 0.001) (Supplementary Material) [30, 31, 33, 37].

Emile and coworkers applied the Apfel score for postoperative nausea and vomiting: they also found a significant improvement with USG-TAP block for this outcome (2.1 ± 0.9 points in the USG-TAP group vs 3.0 ± 0.9 points, *p* < 0.001 in the control group) [35]. Mittal and coworkers reported a pooled number of events of nausea and/or vomiting and found 8/30 and 24/30 cases in the USG-TAP and control groups, respectively [36].

However, both Emile et al. and Saber et al. found that the need for antiemetic use was similar between intervention and control groups [32, 35].

##### Sedation

In the study of Sherif et al., four patients of 47 in the control group required postoperative biphasic intermittent positive airway pressure (BIPAP) ventilation support [33]. According to the study of Sinha et al., four of 50 patients needed BIPAP in the control group [34]. None of these studies detected any need for BIPAP in the USG-TAP group.

Sinha and coworkers also reported significantly lower Richmond Agitation and Sedation Score in the first 6 hours in the USG-TAP block group [34].

##### Time to Ambulate

Pooled analysis of four trials with 347 patients (174 in the intervention group and 173 in the control group) demonstrated that the time to ambulate was shorter by 2.2 h in patients who underwent USG-TAP block (*p* = 0.009) (Fig. 2c) [33–36]. We observed high heterogeneity for this meta-analysis (Fig. 2c). After sensitivity analysis, we identified an influential study [34]. Removal of this study changed the result to non-significant; however, heterogeneity remained high (weighted mean difference (WMD) = − 2.40; 95% CI − 4.98, 0.18; *p* < 0.001 (*p*_heterogeneity_ < 0.001 and *I*^2^ = 96.6%)). (Results of each study are shown in Supplementary Material.)

##### Length of Hospital Stay

A meta-analysis of three studies with 168 patients (83 in the intervention group and 85 in the control group) failed to identify a shorter length of hospital stay following USG-TAP block performance compared with that of controls (*p* = 0.102) (Supplementary Material) [30, 35, 37]. (Results of each study are shown in Supplementary Material.)

##### Length of Operation

Three studies with 121 patients (61 in the intervention group and 60 in the control group) using preoperative USG-TAP block evaluated the length of operation. We found similar operative times in the intervention and control groups (*p* = 0.951) (Supplementary Material) [30–32]. (Results of each study are shown in Supplementary Material.)

##### Satisfaction Rate

Two studies investigated the patient satisfaction rate with different methods. In the study of Mittal and coworkers, it was assessed by the Capuzzo composite score (score range 0–10) in 60 patients: the authors reported significantly higher scores in the USG-TAP block group compared with the control group (8.2 ± 0.7 vs 7.1 ± 0.7; *p* < 0.001) [36]. Sinha and coworkers also observed significantly higher satisfaction scores in the USG-TAP block group at the end of the first postoperative day [34].

##### USG-TAP Block–Related Complications

Only three occurrences of local complications (two cases with hematoma formation, one case with severe pain at the site of injection) due to USG-TAP block were reported in only one study [35].

### Trial Sequential Analysis

The cumulative Z curve crossed trial sequential significance boundary with regard to the outcomes: time to ambulate, nausea and vomiting, pain at 1 and 24 h. In addition, nausea and vomiting and pain at 1 h exceeded the required meta-analysis sample size, from which it can be inferred that inclusion of further clinical trials would not change these results (Fig. S5). TSA for morphine requirement and operation time could not be performed due to insufficient availability of data.

### Risk of Bias in the Studies Included

**Fig. 3:**
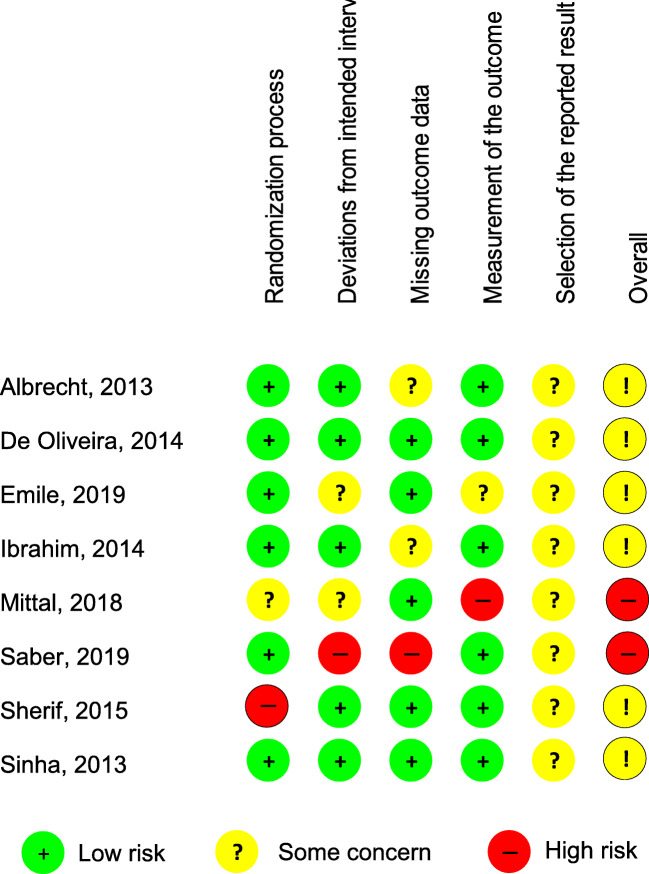
Risk of bias summary: review authors’ judgments about each risk of bias item for each included study

We summarized the results of the risk of bias assessment for each included study in Fig. 3 and Fig. S6.

## Discussion

This meta-analysis and systematic review investigates the efficacy and safety of USG-TAP block compared with systemic analgesia alone in patients undergoing laparoscopic bariatric surgery. Our analyses suggest various beneficial effects, including a reduction in pain scores, in opioid requirement, and in risk for adverse events associated with opioids in the first 24 postoperative hours, without any reported major adverse events.

We detected a statistically significant decrease in resting pain scores at each evaluated time point during the first 24 postoperative hours. Included studies assessed pain intensity by Visual Analog Scale (VAS) or Numbering Rating Scale (NRS) on a scale from 0 to 10. Previously, Kelly and coworkers reported that the minimum clinically significant difference in VAS score is 0.9 [38]. Accordingly, our results may also demonstrate clinically significant improvement, except for the 24th-hour postoperative pain scores, where we found only 0.83 lower WMD in the USG-TAP block group. Two studies also reported a beneficial effect of USG-TAP block on pain 48 h after surgery: the difference between groups was still statistically significant, but it gradually decreased with time [33, 36].

Interestingly, although the mean elimination half-life of bupivacaine is around 8–10 h after USG-TAP block [39], our results hint at a somewhat longer analgesic effectiveness in agreement with previous studies [21, 40], USG-TAP block appears to be effective in late pain as well but to a lesser extent. We evaluated our findings with some reservations because of the low quality of evidence due to inconsistency and the moderate/high risk of bias in individual studies (Table 2).

**Table 2:** Summary of findings table. *Patient or population*: postoperative pain management in obese patients undergoing laparoscopic bariatric surgery; *Setting*: inpatient; *Intervention*: transversus abdominis plane block (TAP block) as a part of multimodal analgesia; *Comparison*: systemic analgesia alone (no intervention or sham-control)

Outcomes	№ of participants (studies) follow-up	Certainty of the evidence (GRADE)	Risk difference with transversus abdominis plane block (TAP block) as a part of multimodal analgesia
Pain score 1 h after surgery assessed with VAS or NRS	347 (4 RCTs)	⨁⨁◯◯ Low^a,b^	MD *2.25 lower* (3.22 lower to 1.28 lower)
Pain score 24 h after surgery assessed with VAS or NRS	347 (4 RCTs)	⨁⨁◯◯ Low^a,b^	MD *0.83 lower* (1.41 lower to 0.24 lower)
24-h postoperative cumulative morphine dose (mg)	118 (3 RCTs)	⨁⨁⨁◯ Moderate^c^	MD *7.59 mg lower* (9.86 lower to 5.32 lower)
Local and systemic complication due to TAP block	525 (8 RCTs)	-	Not pooled
Time to ambulate (h)	347 (4 RCTs)	⨁⨁◯◯ Low^a,b^	MD *2.2 h fewer* (3.89 fewer to 0.56 fewer)

*The risk in the intervention group (and its 95% confidence interval) is based on the assumed risk in the comparison group and the relative effect of the intervention (and its 95% CI)

*CI* confidence interval, *MD* mean difference

GRADE Working Group grades of evidence:

*High certainty*: We are very confident that the true effect lies close to that of the estimate of the effect.

*Moderate certainty*: We are moderately confident in the effect estimate: The true effect is likely to be close to the estimate of the effect, but there is a possibility that it is substantially different.

*Low certainty*: Our confidence in the effect estimate is limited: The true effect may be substantially different from the estimate of the effect.

*Very low certainty*: We have very little confidence in the effect estimate: The true effect is likely to be substantially different from the estimate of effect.

^a^In a single study, there was no information of allocation concealment. In two studies, lack of blinding could lead to bias

^b^Heterogeneity was high for this analysis

^c^Optimal information size is not met calculated by trial sequential analysis

Meta-analysis of four RCTs on cumulative morphine requirement in the first 24 h showed a tendency favoring USG-TAP block, albeit with a high heterogeneity. After removing the influential study verified by sensitivity analysis, heterogeneity disappeared, and the difference became significant.

We speculate that this phenomenon can be due to the much larger intergroup difference in morphine consumption in the influential study compared with the other studies [33]. This might result from the dissimilar study population (predominantly male and leaner patients) and the use of patient-controlled analgesia, unlike in the other trials. It is important to note that although ERAS guidelines recommend patient-controlled administration of opioids, only one study used patient-controlled analgesia (PCA) [9, 33]. We downgraded this outcome to moderate quality of evidence because it was not supported by a large enough data pool (Table 2).

Previous findings in the literature are controversial with regard to the effect of USG-TAP block on morphine requirement. Most studies agree that TAP block reduces opioid requirement in lower [24] and upper abdominal surgeries (as compared with placebo or no intervention) [40]. However, when TAP block was compared with or added to epidural analgesia [41], intrathecal analgesia [42], or wound infiltration in abdominal surgeries [43], there was usually no difference between groups. These findings may suggest that TAP block has no superior or added effect to these techniques in different types of abdominal surgeries. However, some studies have demonstrated the benefits of adding TAP block to infiltration of port sites [44], or even the superiority of TAP block over wound infiltration in general surgery [45].

One of the analyzed studies performed port-site infiltration in both USG-TAP and control groups; this is the only study which did not find significantly reduced morphine consumption in the USG-TAP block group [37]. In contrast, when Ruiz-Tovar and coworkers compared laparoscopic-guided TAP block directly with port-site infiltration in Roux-en-Y gastric bypass surgery, they could demonstrate the superiority of USG-TAP block over port-site infiltration [27]. Based on these findings, it appears that TAP block may lack an added effect to local infiltration anesthesia in bariatric surgery, but it appears to be preferable over local infiltration techniques. Since a definitive conclusion on the comparison of these two methods has not been reached, this topic in both bariatric and other abdominal surgeries would warrant further studies [46].

Enhanced Recovery After Surgery (ERAS) guidelines strongly recommend the administration of multimodal intravenous medication accompanied by local anesthetic infiltration in order to spare or avoid narcotic consumption in a patient group which is highly susceptible to the adverse events of opioids [9]. Nausea, vomiting, constipation, excessive sedation, and respiratory depression may prolong recovery, cause additional complications, and impair satisfaction rate of patients. Previous studies showed that multimodal analgesia reduces the rate of side effects and the time to recovery [47].

Our review discusses thoroughly the effects of USG-TAP block on opioid-related harms; USG-TAP block seems to be beneficial in each evaluated aspect (time to postoperative bowel recovery, nausea and vomiting, sedation). However, we could not reach a strong conclusion based on these results, because the pooled analysis was only possible in the case of nausea and vomiting indicating 76% relative risk reduction, and the 1-h reduction in time to first flatus was on the one hand reported by only one study, and on the other, its clinical relevance is questionable despite the statistically significant result [35].

Our meta-analysis indicates shorter time required to ambulate with USG-TAP block. This may support faster recovery and a reduced number of complications of immobilization. Since both obesity and postoperative conditions are risk factors of thromboembolism, patients with bariatric surgery are at a particularly high risk for these complications [48]. Besides thromboprophylaxis, decreasing length of bed rest can be an important factor in thrombosis prevention. We downgraded this outcome to low quality of evidence because of inconsistency and risk of bias (Table 2).

The presence of USG-TAP block did not affect the total length of hospital stay, even if we would expect that early ambulation would be associated with faster discharge [49]. Nevertheless, since the length of hospital stay depends on several factors, and patients spent only about 2 days in hospital, minor differences might have remained undetected. Further studies assessing the length of hospital stay as the primary outcome could resolve this issue.

TAP block is usually considered safe, but rare complications such as puncture of the liver may occur [50]. Among studies included in this review, only Emile and coworkers reported two cases of abdominal wall hematoma and one case of severe pain at the site of injection [35]. Of course, there are more appropriate study designs to detect rare side effects than RCTs, which could not be included in the current meta-analysis as they did not fit in the inclusion criteria. In the future, it would be important to record complications more thoroughly in RCTs.

Despite the previous concerns regarding challenges to TAP block administration in patients with obesity [51], only two studies mentioned minor difficulties that were successfully eliminated [31, 34]. In addition, we incorporated only those trials that operated under ultrasound guidance, which facilitates better visualization. However, most of the included studies failed to report success rates.

Heterogeneity was high between studies. Since the low number of analyzed studies did not allow subgroup analyses, we were not able to explore the cause of heterogeneity—with one exception mentioned above. Theoretically, we can explain heterogeneity by the different types of surgery, anesthetic management, dose and type of anesthetics, USG-TAP approach, or postoperative analgesia regimen.

It is well known that USG-TAP block relieves somatic but not visceral pain. The ratio of pain types can differ depending on the types of bariatric surgery, affecting the extent of USG-TAP block efficacy, as well. A cadaver study has suggested that the subcostal approach is superior to the mid-axillary approach as indicated by the size of dye spread [52]. In addition, Khan et al. and coworkers achieved better postoperative analgesia with the subcostal approach in patients with cholecystectomy compared with the posterior approach [53]. Thus, the subcostal approach may be better when compared with other techniques in upper abdominal surgeries. It has been also suggested that the pre-incisional application of TAP block may be more potent than post-incisional application, because of the preemptive analgesia that spares patients from the development of altered processing of afferent input [54]. Since we could not perform subgroup analyses to address these questions, further well-designed clinical trials would be required.

In addition to high heterogeneity across studies, the poor reporting of important outcomes by relatively few, small, and single-center studies is another important limitation of our meta-analysis as well as the risk of bias of the included studies. The definition of some outcomes (e.g., operation time) was not precise enough. Conversion of medians to mean could distort our result. Some of the included studies may raise ethical concerns since they worked with invasive placebo (so-called sham-control). The SHAM (serious harm and morbidity) scale classifies the risk of saline injection as placebo control of TAP block as highest (grade 4) [55].

Further limitation can be that some studies were conducted before the “paradigm shift” in opioid use, which means that these studies might apply non-opioids inadequately. The combination of TAP block with non-opioid pain medication within the framework of opioid-restrictive protocols would worth further studying. The analgesic regimens were not only outdated in some studies but also very diverse across studies. For instance, pethidine was used as an opioid in one of the studies, which has become obsolete in several countries for more than two decades [35]. It is, therefore, challenging to compare “old fashioned” single-agent techniques to up-to-date multimodal approaches.

Further studies are also necessary to elucidate the optimal use of USG-TAP block in bariatric surgery, including the ideal timing, technique, dose, or type of local anesthetic injection. We also need to know more about its efficacy when it is added to or compared with other analgesic agents in order to find its place in multimodal analgesia. There are further promising fields in TAP block research as the use of continuous infusion of local anesthetics or liposomal bupivacaine.

## Conclusion

In summary, USG-TAP block reduces pain intensity, morphine requirement, rate of opioid-related side effects, and the time to ambulate. It is likely to help the faster recovery of patients, even if this meta-analysis could not detect significantly shorter length of hospital stay with USG-TAP block. Our results may support its incorporation into multimodal analgesia regimens of ERAS protocols for patients undergoing laparoscopic bariatric surgery, but further studies are needed to evaluate its co-administration with non-opioid medication in opioid-restrictive protocols.

## Electronic Supplementary Material

ESM 1 (PDF 1334 kb).
