# Supplementary material for: Transversus Abdominis Plane Block Appears to Be Effective and Safe as a Part of Multimodal Analgesia in Bariatric Surgery: a Meta-analysis and Systematic Review of Randomized Controlled Trials
Source: Obes Surg. 2020 Oct 21;31(2):531–43. doi: 10.1007/s11695-020-04973-8 (PMC7847866; doi:10.1007/s11695-020-04973-8)

PRISMA 2009 Checklist

Supporting Information Table 1

Section/topic	#	Checklist item	Reported on page #
**TITLE**			
Title	1	Identify the report as a systematic review, meta-analysis, or both.	"Title page" (page 1)
**ABSTRACT**			
Structured summary	2	Provide a structured summary including, as applicable: background; objectives; data sources; study eligibility criteria, participants, and interventions; study appraisal and synthesis methods; results; limitations; conclusions and implications of key findings; systematic review registration number.	"Manuscript" (page 1)
**INTRODUCTION**			
Rationale	3	Describe the rationale for the review in the context of what is already known.	"Manuscript" (page 2)
Objectives	4	Provide an explicit statement of questions being addressed with reference to participants, interventions, comparisons, outcomes, and study design (PICOS).	"Manuscript" (page 2)
**METHODS**			
Protocol and registration	5	Indicate if a review protocol exists, if and where it can be accessed (e.g., Web address), and, if available, provide registration information including registration number.	"Manuscript" (page 3)
Eligibility criteria	6	Specify study characteristics (e.g., PICOS, length of follow-up) and report characteristics (e.g., years considered, language, publication status) used as criteria for eligibility, giving rationale.	"Manuscript" (page 3)
Information sources	7	Describe all information sources (e.g., databases with dates of coverage, contact with study authors to identify additional studies) in the search and date last searched.	"Manuscript" (page 3)
Search	8	Present full electronic search strategy for at least one database, including any limits used, such that it could be repeated.	"Manuscript" (page 3)
Study selection	9	State the process for selecting studies (i.e., screening, eligibility, included in systematic review, and, if applicable, included in the meta-analysis).	"Manuscript" (page 3)
Data collection process	10	Describe method of data extraction from reports (e.g., piloted forms, independently, in duplicate) and any processes for obtaining and confirming data from investigators.	"Manuscript" (page 3)
Data items	11	List and define all variables for which data were sought (e.g., PICOS, funding sources) and any assumptions and simplifications made.	"Manuscript" (page 3)
Risk of bias in individual studies	12	Describe methods used for assessing risk of bias of individual studies (including specification of whether this was done at the study or outcome level), and how this information is to be used in any data synthesis.	"Manuscript" (page 4)

PRISMA 2009 Checklist

Summary measures	13	State the principal summary measures (e.g., risk ratio, difference in means).	"Manuscript" (page 4)
Synthesis of results	14	Describe the methods of handling data and combining results of studies, if done, including measures of consistency (e.g., I^2) for each meta-analysis.	"Manuscript" (page 4)

Risk of bias across studies	15	Specify any assessment of risk of bias that may affect the cumulative evidence (e.g., publication bias, selective reporting within studies).	"Manuscript" (page 4)
Additional analyses	16	Describe methods of additional analyses (e.g., sensitivity or subgroup analyses, meta-regression), if done, indicating which were pre-specified.	"Manuscript" (page 4)
**RESULTS**			
Study selection	17	Give numbers of studies screened, assessed for eligibility, and included in the review, with reasons for exclusions at each stage, ideally with a flow diagram.	"Manuscript" (page 5), Figure 1
Study characteristics	18	For each study, present characteristics for which data were extracted (e.g., study size, PICOS, follow-up period) and provide the citations.	"Manuscript" (page 5), Table 1
Risk of bias within studies	19	Present data on risk of bias of each study and, if available, any outcome level assessment (see item 12).	"Manuscript" (page 8), Figure 3
Results of individual studies	20	For all outcomes considered (benefits or harms), present, for each study: (a) simple summary data for each intervention group (b) effect estimates and confidence intervals, ideally with a forest plot.	"Manuscript" (page 4–8), Figure 2, Suppl. Figure 2, 3, 4
Synthesis of results	21	Present the main results of the review. If meta-analyses are done, include for each, confidence intervals and measures of consistency	"Manuscript" (page 4–8), Figure 2, Suppl. Figure 2, 3, 4
Risk of bias across studies	22	Present results of any assessment of risk of bias across studies (see Item 15).	"Manuscript" (page 8), Supplemental Figure 6

PRISMA 2009 Checklist

Additional analysis	23	Give results of additional analyses, if done (e.g., sensitivity or subgroup analyses, meta-regression [see Item 16]).	"Manuscript" (page 4–8), Suppl. Figure 5
**DISCUSSION**			
Summary of evidence	24	Summarize the main findings including the strength of evidence for each main outcome; consider their relevance to key groups (e.g., healthcare providers, users, and policy makers).	"Manuscript" (page 8–10)
Limitations	25	Discuss limitations at study and outcome level (e.g., risk of bias), and at review-level (e.g., incomplete retrieval of identified research, reporting bias).	"Manuscript" (page 11)
Conclusions	26	Provide a general interpretation of the results in the context of other evidence, and implications for future research.	"Manuscript" (page 11)
**FUNDING**			
Funding	27	Describe sources of funding for the systematic review and other support (e.g., supply of data); role of funders for the systematic review.	"Manuscript" (page 12)

From: Moher D, Liberati A, Tetzlaff J, Altman DG, The PRISMA Group (2009). Preferred Reporting Items for Systematic Reviews and Meta-Analyses: The PRISMA Statement. PLoS Med 6(7): e1000097. doi:10.1371/journal.pmed1000097

For more information, visit: **www.prisma-statement.org**.

Supporting Information Table 2. Study-level data in each study

Study name	mean (SD) or median (Q1-Q3) or mean (CI 95%) in USG-TAP group	mean (SD) or median (Q1-Q3) or (CI 95%) in control group	p-value
24-hour opioid requirement (mg)			
Albrect, 2013	32.2 (CI 27.6-36.7)	35.6 (CI 28.6-42.5)	0.41
De Oliveira, 2014	7.5 (2.5-11.5)	13.0 (7.0-21.5)	0.07
Ibrahim, 2013	16.76 (2.7)	24.76 (5.0)	<0.001
Time to ambulate (h)			
Emile, 2019	6.3 (1.0)	7.3 (1.2)	<0.001
Mittal, 2018	8.2 (2.3)	9.5 (2.5)	0.045
Sinha, 2013	6.3 (1.8)	8.0 (1.8)	<0.001
Sherif, 2015	6.85 (1.8)	11.8 (2.6)	<0.001
Length of hospital stay (h)			
Albrecht, 2013	56.1 (47.8-64.4)	50.2 (45.7-54.6)	0.19
De Oliveira, 2014	32.1 (13.9-52.6)	22.5 (19.0-26.0)	0.47
Emile, 2019	42.24 (13.4)	42.24 (13.4)	0.87
Length of operation (min)			
De Oliveira, 2014	76.5 (51-106)	92 (61-120)	0.53
Ibrahim, 2013	119.3 (10.4)	120.6 (13.3)	0.293
Saber, 2019	56.4 (13.5)	54.8 (17.5)	0.393

Supporting Information Figure 1

Search strategy in detail

Database: CENTRAL

Date of search: 20.09.2019

Number of records: 89

(bariatric* OR “bariatric surgery” OR “metabolic surgery” OR “weight loss surgery” OR “sleeve gastrectomy” OR “gastric bypass” OR “gastric band*” OR “biliopancreatic diversion” OR “duodenal switch” OR “omega switch” OR “vertical banded gastroplasty” OR “sleeve resection” OR ”jejunoileal bypass” OR ”banded gastroplast*”) AND (TAP OR "plane block" "abdominal transverse" OR "transversus abdominis" OR regional an*esthe* OR regional analg*)

Database: MEDLINE

Date of search: 20.09.2019

Number of records: 36

(bariatric* OR “bariatric surgery” OR “metabolic surgery” OR “weight loss surgery” OR “sleeve gastrectomy” OR “gastric bypass” OR “gastric band*” OR “biliopancreatic diversion” OR “duodenal switch” OR “omega switch” OR “vertical banded gastroplasty” OR “sleeve resection” OR ”jejunoileal bypass” OR ”banded gastroplast*”) AND (TAP OR "plane block" "abdominal transverse" OR "transversus abdominis" OR regional an*esthe* OR regional analg*)

Database: Web of Science

Date of search: 20.09.2019

Number of records: 99

(bariatric* OR “bariatric surgery” OR “metabolic surgery” OR “weight loss surgery” OR “sleeve gastrectomy” OR “gastric bypass” OR “gastric band*” OR “biliopancreatic diversion” OR “duodenal switch” OR “omega switch” OR “vertical banded gastroplasty” OR “sleeve resection” OR ”jejunoileal bypass” OR ”banded gastroplast*”) AND (TAP OR "plane block" "abdominal transverse" OR "transversus abdominis" OR regional an*esthe* OR regional analg*)

Database: Embase

Date of search: 20.09.2019

Number of records: 127

(bariatric* OR “bariatric surgery” OR “metabolic surgery” OR “weight loss surgery” OR “sleeve gastrectomy” OR “gastric bypass” OR “gastric band*” OR “biliopancreatic diversion” OR “duodenal switch” OR “omega switch” OR “vertical banded gastroplasty” OR “sleeve resection” OR ”jejunoileal bypass” OR ”banded gastroplast*”) AND (TAP OR "plane block" "abdominal transverse" OR "transversus abdominis" OR regional an*esthe* OR regional analg*)

Supporting Information Figure 2. Forest plot showing postoperative nausea

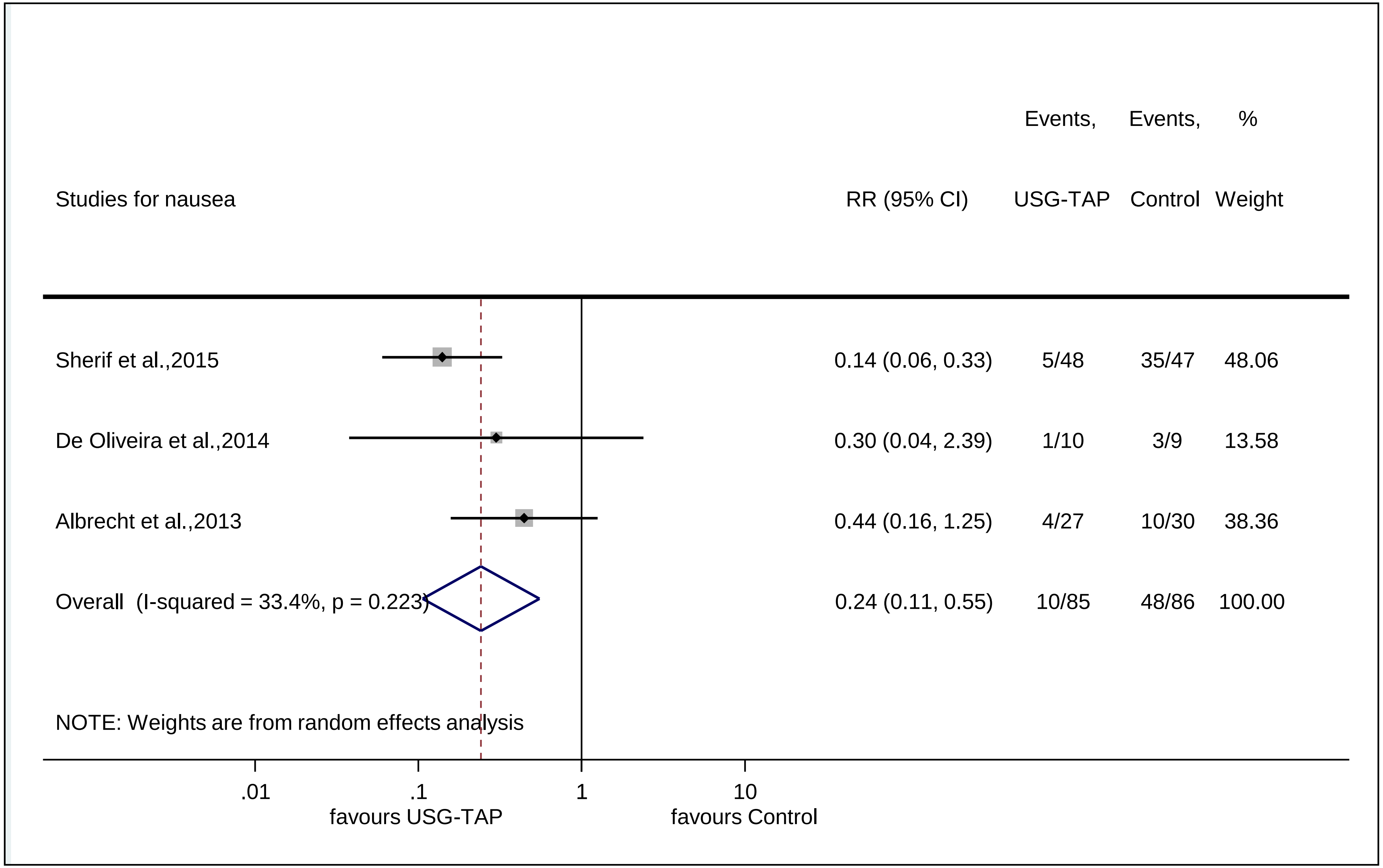

Supporting Information Figure 3. Forest plot showing length of hospital stay (hours)

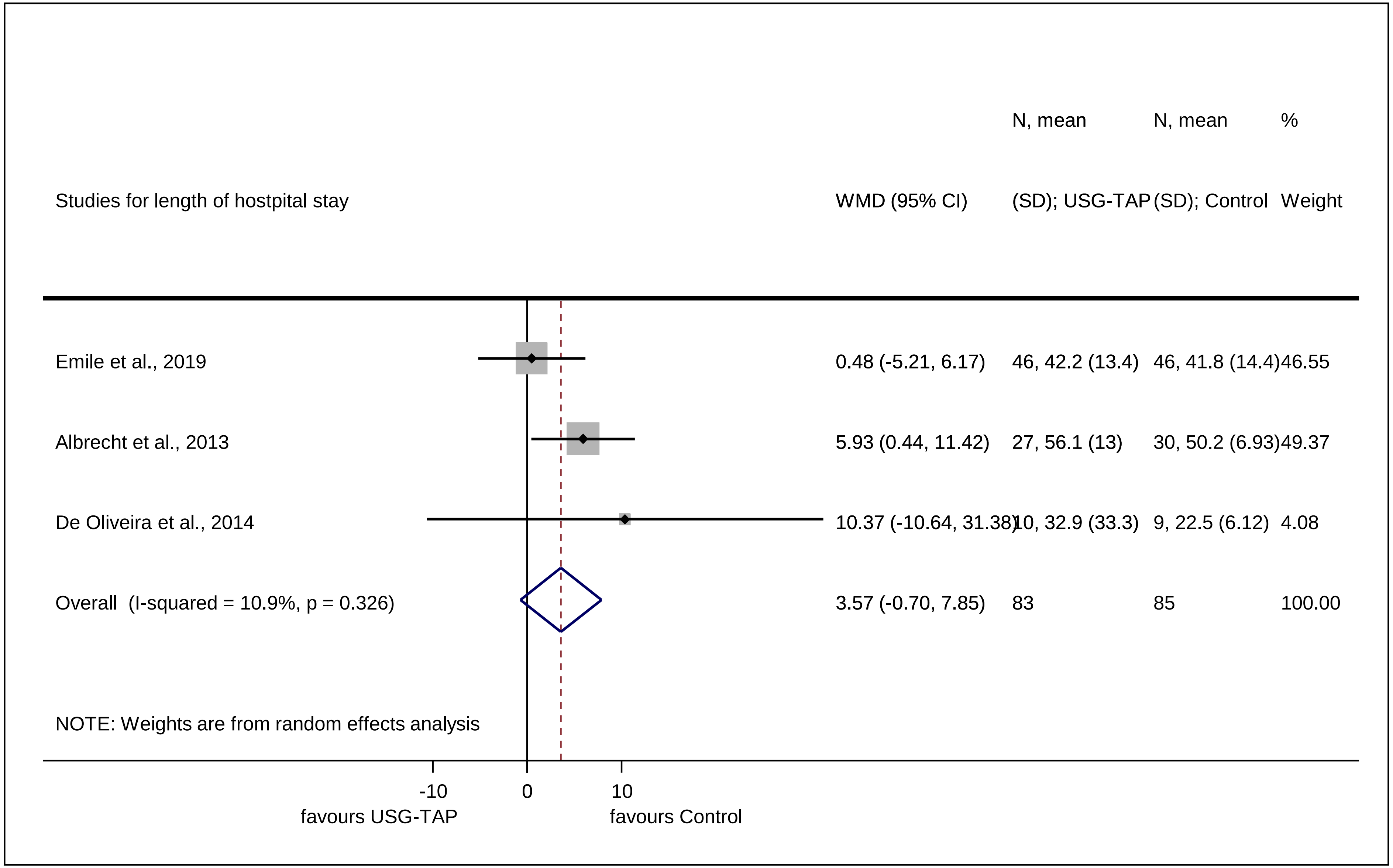

Supporting Information Figure 4. Forest plot showing operation time (hours)

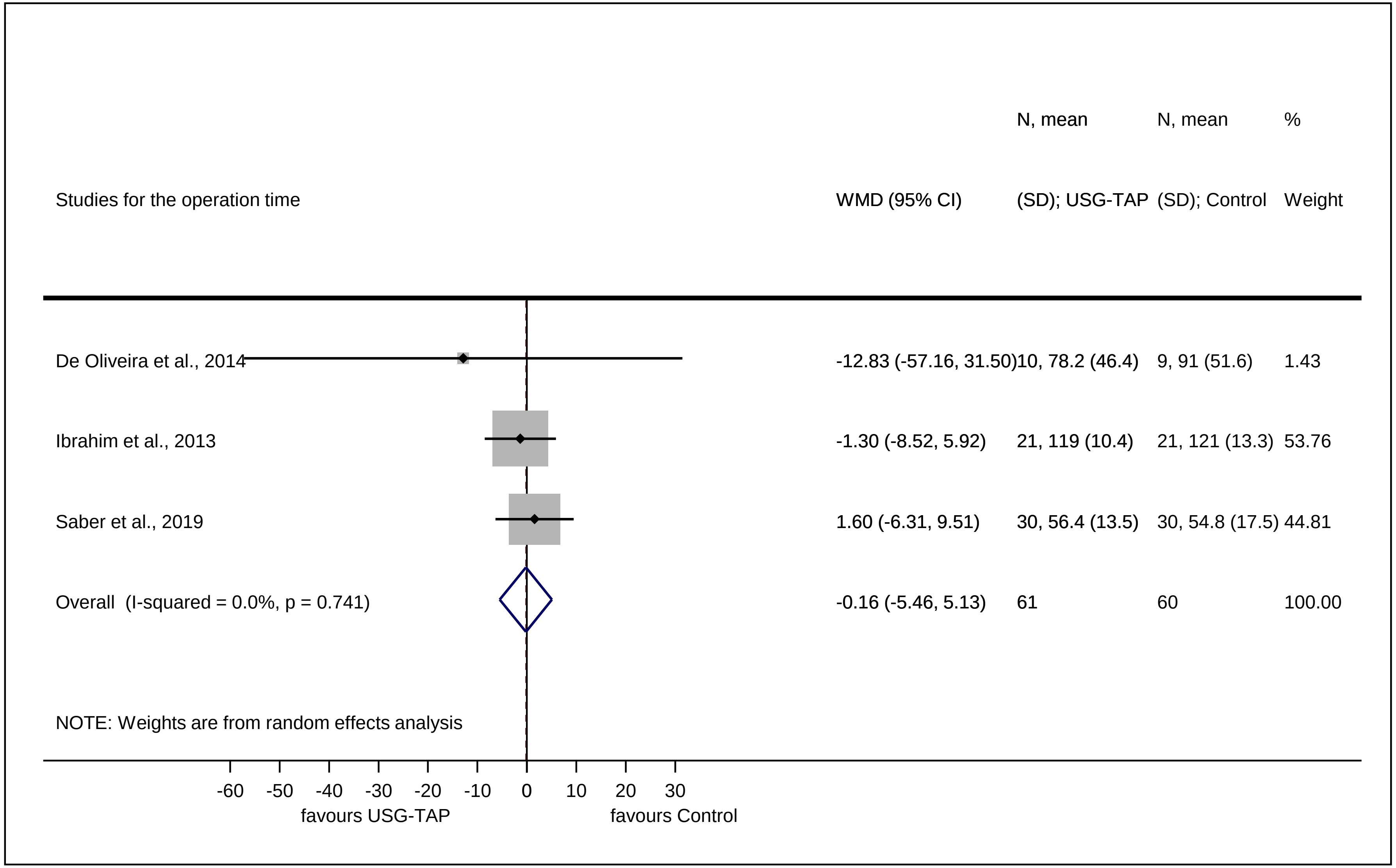

Supporting Information Figure 5. Trial sequential analyses (TSA) for efficacy endpoints

A TSA for time to ambulate (hours)

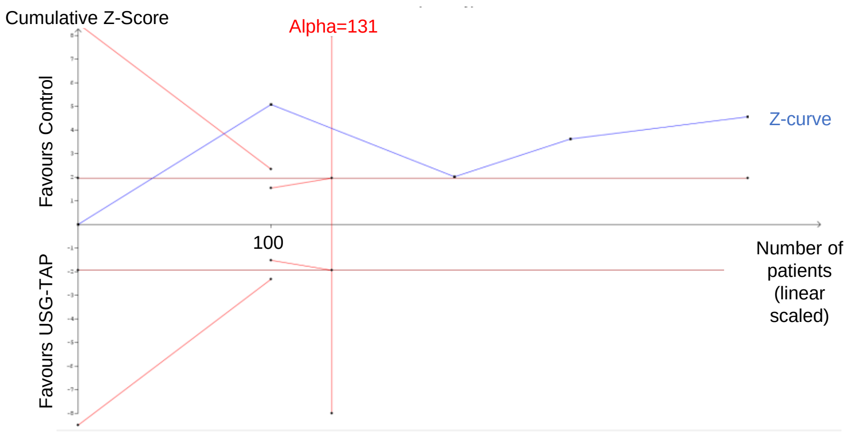

B TSA for nausea

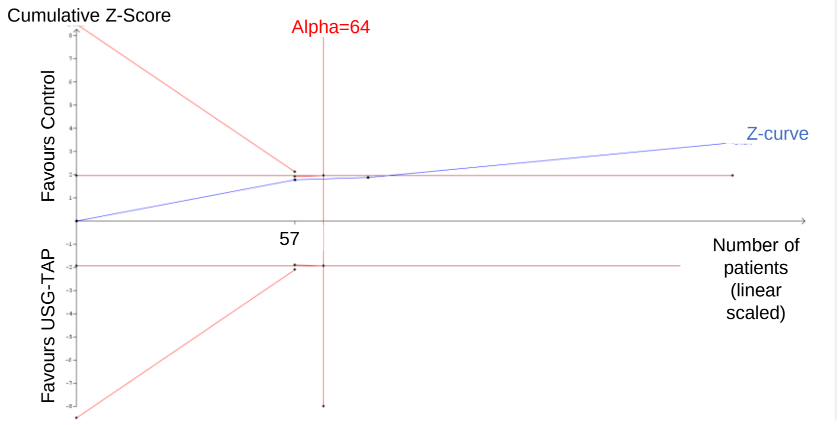

C TSA for 1-hour postoperative pain score

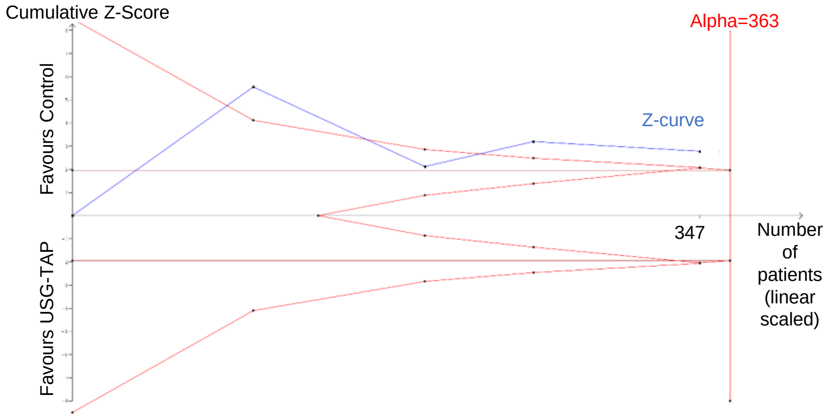

D TSA for 24-hour pain postoperative score

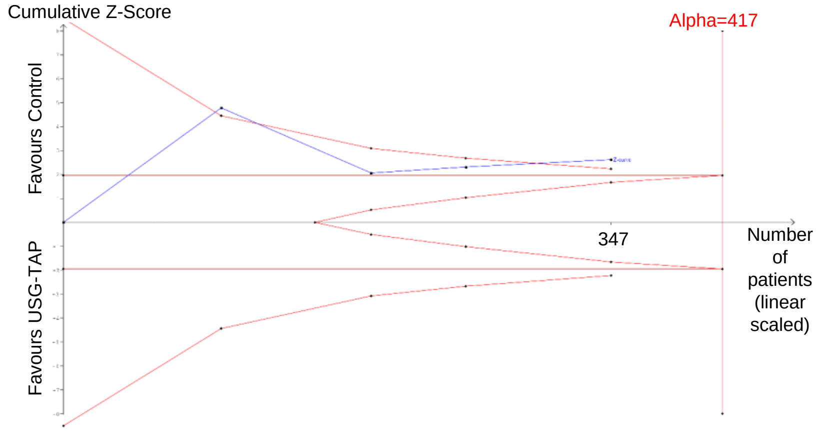

Supporting Information Figure 6. Risk of bias across studies

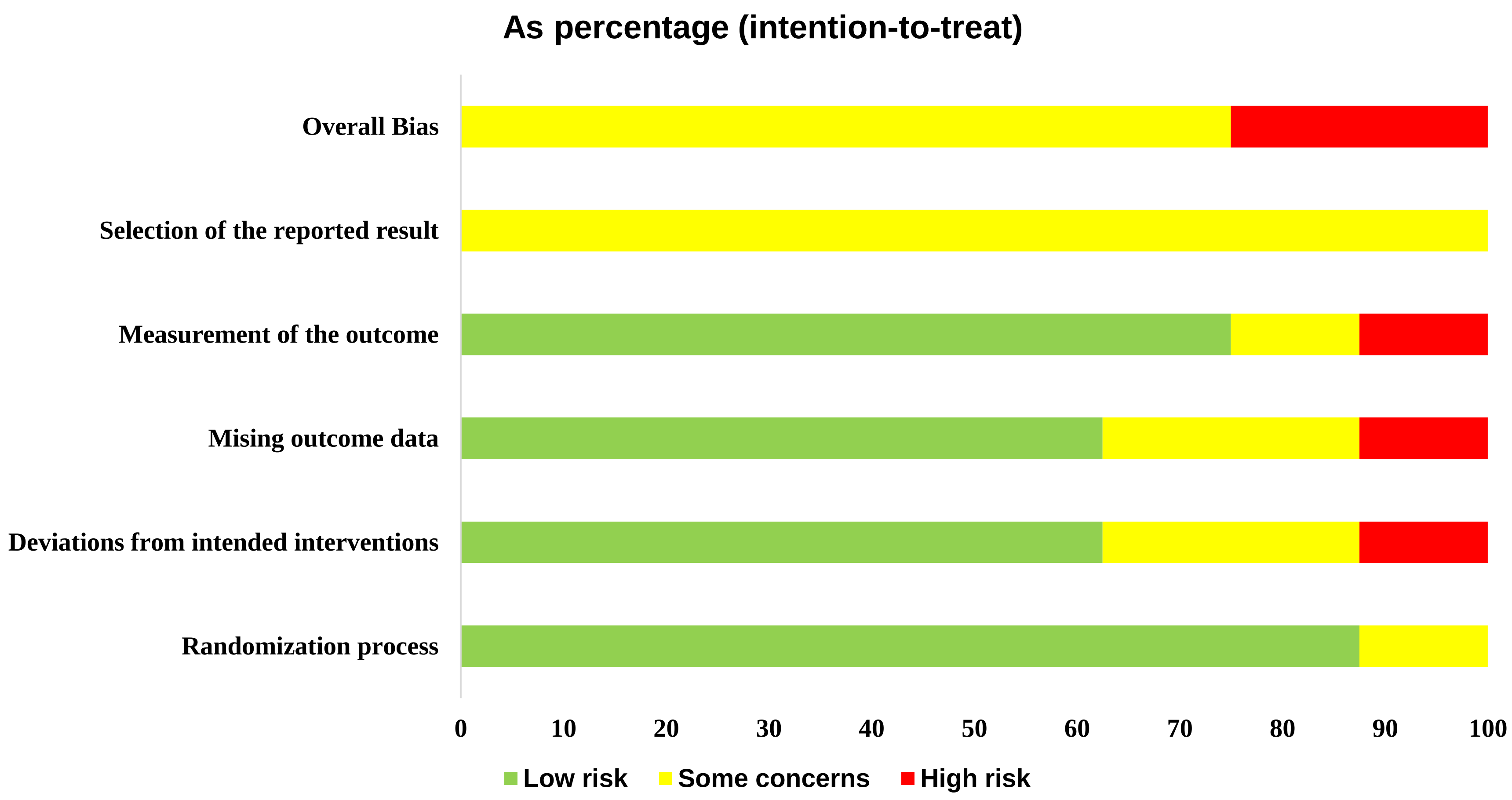

Supplement: Supplementary file 1 — (PDF 1334 kb). [file 11695_2020_4973_MOESM1_ESM.pdf]
